# Deep learning-enhanced super-resolution diffusion-weighted liver MRI: improved image quality, diagnostic performance, and acceleration

**DOI:** 10.1186/s13244-025-02150-y

**Published:** 2025-12-08

**Authors:** Dan Zhao, Xiangchuang Kong, Kun Yang, Jiayu Wan, Ziyi Liu, Feng Pan, Peng Sun, Chuansheng Zheng, Lian Yang

**Affiliations:** 1 Department of Radiology, Union Hospital, Tongji Medical College, Huazhong University of Science and Technology, 430022 Wuhan, Hubei China; 2 Hubei Provincial Clinical Research Center for Precision Radiology & Interventional Medicine, 430022 Wuhan, Hubei China; 3 Hubei Province Key Laboratory of Molecular Imaging, 430022 Wuhan, Hubei China; 4 Clinical and Technical Support, Philips Healthcare, 100600 Beijing, China

**Keywords:** Deep learning reconstruction, Diffusion-weighted imaging, Apparent diffusion coefficient, Liver MRI, Focal liver lesions

## Abstract

**Objectives:**

To investigate the impact of deep learning reconstruction (DLR) on the image quality of diffusion-weighted imaging (DWI) for liver and its ability to differentiate benign from malignant focal liver lesions (FLLs).

**Materials and methods:**

Consecutive patients with suspected liver disease who underwent liver MRI between January and May 2025 were included. All patients received conventional DWI (DWI_C_) and an accelerated reconstructed DWI (DWI_DLR_) in which acquisition time was prospectively halved by reducing signal averages. Image quality was compared qualitatively using Likert scores (e.g., lesion conspicuity, overall quality) and quantitatively by measuring signal-to-noise ratio of the liver (SNR_Liver_) and lesion (SNR_Lesion_), contrast-to-noise ratio (CNR), and edge rise distance (ERD). Apparent diffusion coefficient (ADC) values and diagnostic performance for differentiating benign from malignant FLLs were assessed.

**Results:**

A total of 193 patients (128 males, 65 females; age range, 23-81 years) were included. For quantitative assessment, DWI_DLR_ demonstrated higher SNR_Liver_, SNR_Lesion_, CNR, and a shorter ERD (all *p* < 0.05). For qualitative assessment, DWI_DLR_ showed improved lesion conspicuity, liver edge sharpness, and overall image quality (all *p* < 0.01), with no significant difference in artifacts (*p* = 0.08). ADC values were lower with DWI_DLR_ for both benign and malignant FLLs (*p* < 0.001). In differentiating benign from malignant lesions, DWI_DLR_ achieved better diagnostic performance (AUC: 0.921 vs. 0.904, *p* < 0.05).

**Conclusion:**

Deep learning-enhanced DWI enables a 50% reduction in acquisition time while simultaneously improving liver MRI image quality and diagnostic performance in differentiating benign from malignant FLLs.

**Critical relevance statement:**

This study demonstrates that deep learning-based reconstruction enables faster, higher-quality liver MRI with improved diagnostic accuracy for focal liver lesions, supporting its integration into routine radiological practice.

**Key Points:**

- Diffusion-weighted liver MRI commonly suffers from limited image quality and efficiency.
- Deep learning reconstruction substantially improves liver MRI quality while enabling significantly shorter acquisition times.
- Improved lesion differentiation enables more accurate clinical diagnosis of liver lesions.

**Graphical Abstract:**

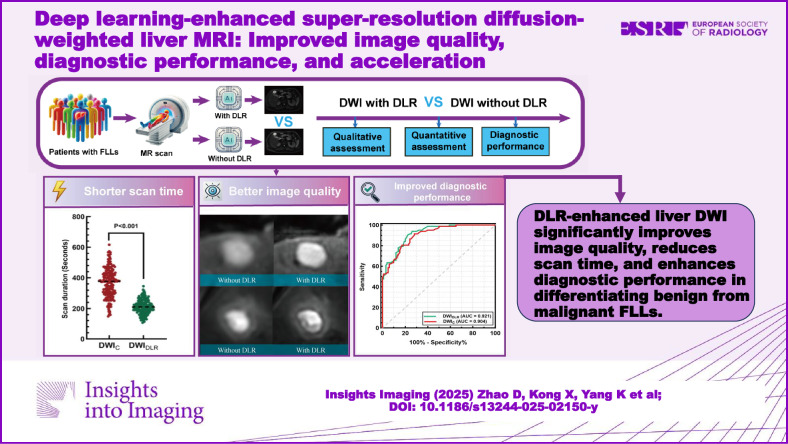

## Introduction

Currently, diffusion-weighted imaging (DWI) is widely used in liver imaging, playing a crucial role in the detection and characterization of hepatic lesions [1]. DWI reflects tissue cellularity through water molecule diffusion [2]. The degree of water molecule diffusion is quantified by the apparent diffusion coefficient (ADC), which has been shown to help distinguish between benign and malignant lesions [3]. Additionally, DWI and ADC measurements are increasingly used to evaluate tumor response to treatment [4]. Due to its high sensitivity in liver lesion detection and differentiation [1, 5], DWI has now been widely integrated into routine clinical practice, demonstrating significant benefits in evaluating focal hepatic tumors [6].

However, achieving high and consistent diagnostic quality in clinical practice remains challenging due to the limitations of DWI image quality, especially for liver imaging, which is subject to respiratory motion, heartbeat, and gastrointestinal movement [7]. Additionally, the image quality of DWI is influenced by the *b*-value. While higher *b*-values are crucial for characterizing liver lesions and increasing specificity for malignant lesions [8], they lead to poorer image quality and reduced reproducibility [9, 10].

Several techniques, including parallel imaging (PI), compressed sensing (CS), and respiratory-triggering, have been introduced to improve DWI image quality [11–13]. Although PI and CS aim to accelerate scans, they can introduce reduced signal-to-noise ratio (SNR) and residual artifacts. Respiratory-triggering, while effective for motion control, inherently prolongs scan time [14]. Recent advancements in deep learning reconstruction (DLR) have shown promise in overcoming these challenges, improving DWI image quality in domains such as prostate, brain, and musculoskeletal imaging [15–17]. It has also been applied to liver DWI, which was reported to improve the detection rate of liver lesions [18], image quality [19], and scanning efficiency [20]. However, previous applications of DLR in liver DWI have primarily focused on denoising and acceleration rather than dedicated super-resolution reconstruction. This may limit the potential for improving spatial resolution and the clarity of fine anatomical details. Additionally, research is lacking on whether applying DLR in DWI can enhance diagnostic performance for focal liver lesions (FLLs).

Herein, we employed a novel deep learning algorithm for liver DWI sequences, consisting of k-space reconstruction, image denoising, and resolution enhancement, aiming to improve image quality and reduce scan time. We assessed the image quality of DWI both qualitatively and quantitatively, comparing deep learning reconstructed DWI (DWI_DLR_) to conventional DWI (DWI_C_). Furthermore, we aimed to evaluate their diagnostic performance in distinguishing benign from malignant FLLs using ADC values.

## Material and methods

### Patients

**Fig. 1:**
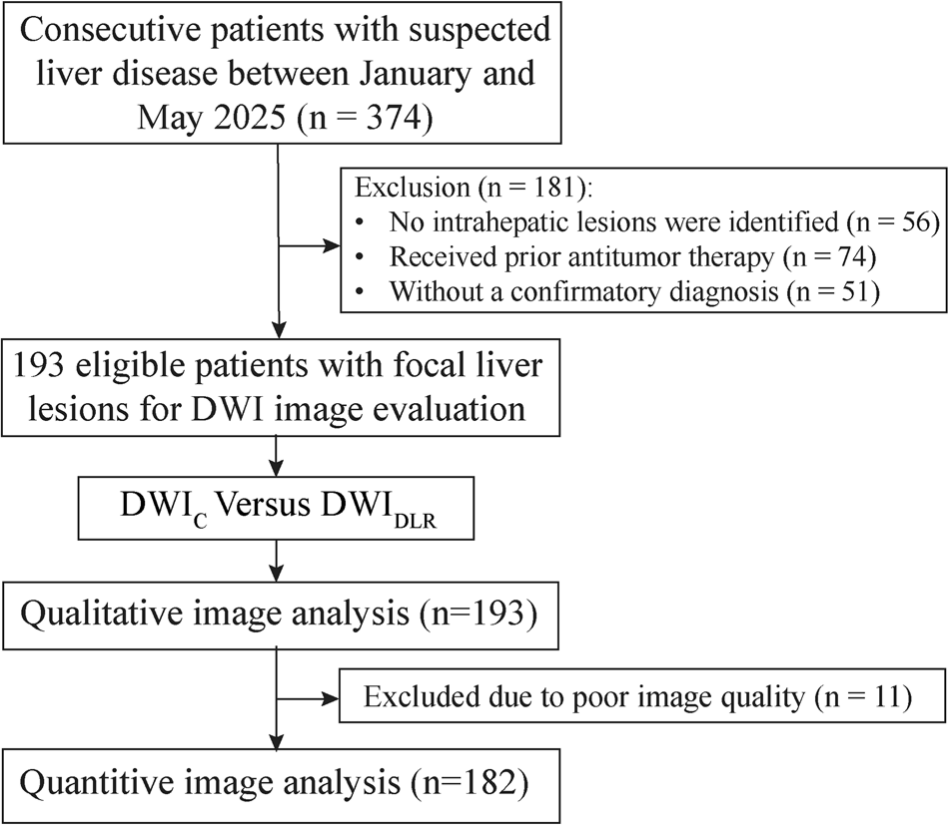
Flow chart illustrating patient selection and study flow. DWI_C_, conventional diffusion-weighted imaging; DWI_DLR_, deep learning reconstructed diffusion-weighted imaging

This prospective study was approved by the institutional review board of Wuhan Union Hospital (Approval number: No.2025-0666), and written informed consent was obtained from all patients. Consecutive patients with suspected liver disease (including elevated tumor marker, elevated liver enzyme, known malignancy outside the liver, hepatic lesions found on ultrasound or CT, or extrahepatic malignancy) who underwent DWI_C_ and DWI_DLR_ were included in this study from January to May 2025. Exclusion criteria include (a) no intrahepatic lesions were identified; (b) received prior antitumor therapy; (c) without a confirmatory diagnosis. The flow chart is shown in Fig. 1.

### Liver MRI

Liver MRI was performed at 3.0 T (Ingenia Elition X, Philips Healthcare) using a 32-channel phased-array coil. The sequences included DWI_C_, DWI_DLR_, T2-weighted fast spin-echo images, and a dynamic contrast-enhanced T1-weighted fast field echo sequence with a modified dual-echo 2-point Dixon technique [21]. A respiratory-triggered fat-suppressed single-shot echoplanar DWI sequence was performed in the transverse plane with tri-directional diffusion gradients using two *b*-values: 0 and 800 s/mm^2^. The use of two *b*-values for mono-exponential ADC calculation was chosen as this approach represents a widely accepted approach in clinical hepatic imaging to provide sufficient diffusion contrast for lesion characterization [6]. For DWI_C_, we used two averages to ensure sufficient image quality, while for DWI_DLR_, only one excitation was used, halving the scanning time. This resulted in a mean acquisition time of 212.3 ± 46.7 s for DWI_DLR_, compared to 369.1 ± 96.6 s for DWI_C_ (*p* < 0.001), as detailed in Fig. S3. Both sequences employed identical respiratory-triggering schemes to mitigate potential motion artifacts. Gadobutrol (Gadovist, Bayer Schering Pharma) was administered for contrast-enhanced T1-weighted sequences. Acquisition protocol parameters are listed in Table S1.

The deep learning super-resolution algorithm was provided by Philips Healthcare, which aims to accelerate scanning and enhance image resolution. Briefly, the advanced deep learning framework integrates compressed sensing with two specialized convolutional neural networks (CNNs). The first network (Adaptive-CS-Net) was designed for sparsity-constrained reconstruction with nonuniform random subsampling, based on established methodologies [22, 23]. The second network (SuperRes-Net) is employed for the triple purpose of denoising, eliminating ringing artifacts, and enhancing image resolution [24, 25]. Both sequences had a base resolution of 132 × 108 mm. Resolution in DWI_C_ images was increased to 352 ×  288 mm using interpolation, whereas DWI_DLR_ images achieved a resolution of 352 × 288 mm through deep neural networks. The deep learning framework is detailed in the supplementary materials and Fig. S1.

### Image analysis

MR images were transferred to a workstation (IntelliSpace Portal version 12.1, Philips Healthcare) for postprocessing. For patients with multiple lesions, the target lesion prioritized for follow-up or treatment was selected for image analysis.

The reference standard for detecting and characterizing FLLs was established by consensus between two radiologists (P.F. and Y.L., with 15 and 25 years of experience in abdominal imaging, respectively). This standard relied on typical MR imaging findings confirmed by clinical history, pathology, and follow-up imaging. Benign lesions, including hepatic hemangioma (HH), focal nodular hyperplasia (FNH), adenoma, abscess, and cyst, were diagnosed using established criteria [26], and their stability on follow-up MR imaging. Diagnoses of malignant lesions, including hepatocellular carcinoma (HCC), cholangiocarcinoma (CCA), and liver metastases (LMs), were based on clinical history, MR imaging features [27], pathology, and interval progression on follow-up imaging.

For qualitative analysis of the DWI images, another two radiologists (Z.L. and X.K., with 5 and 10 years of experience in abdominal imaging, respectively), who routinely use DWI_C_ in their daily clinical practice, independently reviewed the two data sets (DWI_C_ and DWI_DLR_) in a randomized and blinded fashion. Four categories, including overall image quality, sharpness of vessels, artifacts, and lesion conspicuity, were assessed based on a 5-point Likert scale (1, non-diagnostic; 2, poor; 3, moderate; 4, good; 5, excellent), with detailed definitions provided in Table S2. To minimize inter-reader variability and standardize their evaluation approach, they underwent a training session with 20 sample cases. If the scores differed, the radiologists discussed and reached a consensus.

For the quantitative evaluation of the DWI images, regions-of-interest (ROI) measuring 100-300 mm² were placed in the liver parenchyma (right and left lobes), carefully avoiding intrahepatic vessels and bile ducts and maintaining a distance of at least 5 mm from the liver edge. For liver lesions, the ROI was delineated along the maximum diameter of the lesion, avoiding the edges. To mitigate the potential influence of DLR on background noise, an ROI was placed on paravertebral muscles, and their standard deviation was used as a measure of “intra-noise” [19].

The signal-to-noise ratio (SNR) and contrast-to-noise ratio (CNR) were calculated as follows:

$${{{{\rm{SNR}}}}}_{{{{\rm{Liver}}}}}=\frac{{{{{\rm{SI}}}}}_{{{{\rm{Liver}}}}}}{{{{{\rm{SD}}}}}_{{{{\rm{paravertebral}}}}\; {{{\rm{muscles}}}}}}$$

$${{{{\rm{SNR}}}}}_{{{{\rm{Lesion}}}}}=\frac{{{{{\rm{SI}}}}}_{{{{\rm{Lesion}}}}}}{{{{{\rm{SD}}}}}_{{{{\rm{paravertebral}}}}\; {{{\rm{muscles}}}}}}$$

$${{{\rm{CNR}}}}={{{{\rm{SNR}}}}}_{{{{\rm{Lesions}}}}}-{{{{\rm{SNR}}}}}_{{{{\rm{Liver}}}}}$$

where ${{{{\rm{SI}}}}}_{{{{\rm{Liver}}}}}$, ${{{{\rm{SI}}}}}_{{{{\rm{Lesion}}}}}$ represent the averaged signal intensity of normal liver and lesion, respectively, ${{{{\rm{SD}}}}}_{{{{\rm{paravertebral\; muscles}}}}}$ is the averaged standard deviation of the paravertebral muscles. The ROI setting is shown in Fig. S2.

**Fig. 2:**
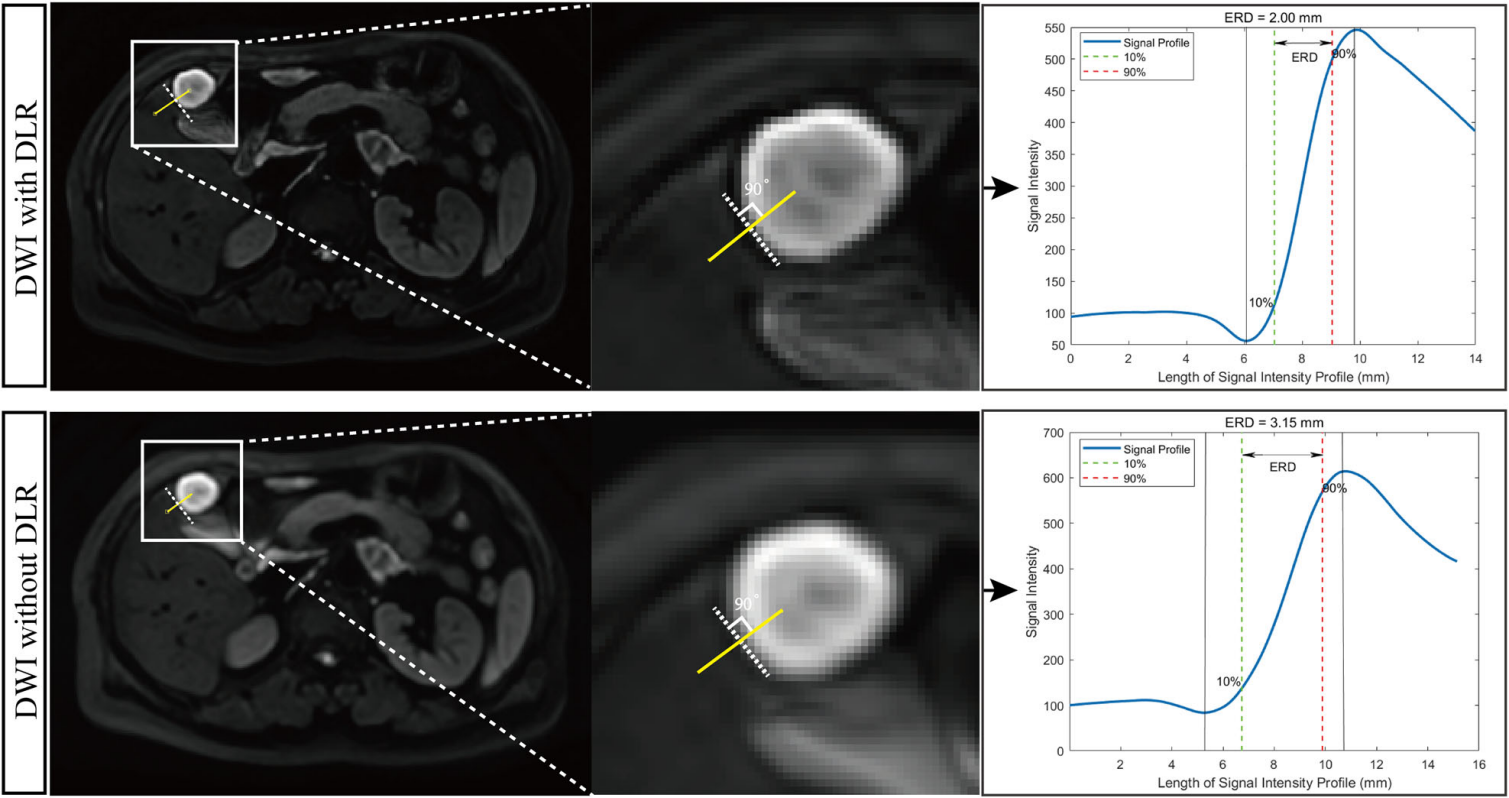
ERD of liver MRI with or without DLR. Lesion boundary sharpness was assessed by drawing a short line segment (yellow) from normal liver tissue into the lesion, perpendicular to the tangent at the lesion edge. This line segment was used to obtain the signal intensity profile and calculate the ERD. A shorter ERD indicates a sharper lesion boundary. The figure shows that DWI with deep learning reconstruction provides superior sharpness compared to DWI without DLR. ERD, edge rise distance

The sharpness of the lesion was evaluated based on edge rise distance (ERD) [28]. Briefly, a line perpendicular to the lesion border was drawn from the lesion to normal liver tissue. The signal intensity profiles were established along the line segment, averaged, and plotted. ERD was defined as the distance between 10% and 90% of the signal intensity level of lesions (Fig. 2). The measurements were performed by Matrix Laboratory (MATLAB, version R2023b).

For ADC measurement, pixel-based ADC maps were obtained at two *b*-values (*b* = 0 and 800 s/mm^2^). The same two radiologists (Z.L. and X.K.) measured the mean ADC of each lesion by drawing an ROI over the largest cross-sectional area of the lesion. To ensure consistency, the ROI were placed in the DWI images and then copied into the ADC maps. The mean ADC values measured by two radiologists were used as representative values. For lesions not well visualized on DW images, location was determined using postcontrast T1-weighted images.

### Statistical analysis

Quantitative evaluation of DWI images (SNR_Liver_, SNR_Lesion_, CNR, ERD) and ADC value was compared by using paired *t*-tests, and qualitative analysis (5-point Likert scale) was compared using the Wilcoxon signed-rank test.

Agreement between the two different DWI imaging techniques for measuring ADC values across all lesions was assessed using Bland-Altman analysis. To further evaluate the influence of imaging technique and lesion type on ADC measurements, a two-way ANOVA was performed, with imaging techniques (DWI_C_ vs. DWI_DLR_) and lesion types as fixed factors. To assess the diagnostic performance of DWI_C_ vs. DWI_DLR_ in differentiating benign from malignant FLLs based on ADC values, receiver operating characteristic (ROC) analyses were conducted. Additionally, to address the potential confounding effect of high-ADC lesions (cysts and abscesses), a specific sub-analysis was performed to evaluate the diagnostic performance in differentiating malignant lesions from the most common solid benign lesions, FNH and HH, combined. The ADC threshold value was determined based on the Youden index. The areas under the ROC curves (AUCs) were compared using the DeLong method.

Interobserver agreement for qualitative scoring was not calculated as scores were determined by consensus, eliminating initial observer discrepancies. For the interobserver agreement of quantitative metrics (SNR_Liver_, SNR_Lesion_, CNR, ERD, and ADC), the intraclass correlation coefficient (ICC) was used (< 0.5 = poor; 0.5 to < 0.75 = moderate; 0.75 to < 0.9 = good; ≥ 0.9 = excellent).

A *p*-value < 0.05 was considered statistically significant. All statistical analyses were performed with SPSS Version 22.0 (SPSS Inc.).

## Results

### Clinical characteristics

A total of 374 patients with suspected liver disease who underwent liver MRI were initially included. Among them, 181 patients were excluded based on the exclusion criteria (Fig. 1). Ultimately, 193 patients (128 men, 65 women; mean age, 56.8 years; age range, 23-81 years) were included. One-hundred and five patients had malignant solid FLLs, including 53 HCCs, 33 LMs, and 19 CCAs. Of these, 42 HCCs, 16 LMs, and 12 CCAs were pathologically confirmed, while the other malignant lesions were clinically diagnosed. Eighty-eight patients had benign lesions, including 38 HHs, 19 FNHs, 10 hepatic adenomas, 13 liver abscesses, and 8 liver cysts. Of these, two hepatic adenomas were diagnosed pathologically. The diagnosis of the remaining benign lesions was determined clinically according to the reference standard. The characteristics of the patients and lesions are summarized in Table 1.

**Table 1:** Characteristics of patients and focal liver lesions

Patient characteristics	Value
Mean age (age range)	56.8 ± 16.9 (23–81)
Sex	
Male	128 (66.3)
Female	65 (33.7)
Background liver	
Chronic hepatitis	76 (39.4)
Liver cirrhosis	49 (25.4)
Steatosis	21 (10.9)
Normal liver	85 (44.0)
FLL characteristics	
FLL size (cm)	3.2 ± 2.0
Benign	3.4 ± 1.9
Malignant	3.1 ± 2.1
FLL location	193
Right lobe	126 (65.3)
Left lobe	67 (34.7)
Diagnosis of the FLL	193
Benign	88 (45.6)
HH	38 (19.7)
FNH	19 (9.8)
Hepatic adenomas	10 (5.2)
Liver abscesses	13 (6.7)
Liver cysts	8 (4.1)
Malignant	105 (54.4)
HCC	53 (27.5)
Liver metastases	33 (17.1)
Cholangiocarcinoma	19 (9.8)

Data are shown as mean ± standard deviation or number (percentage)

*FLL* focal liver lesions, *HH* hepatic hemangioma, *FNH* focal nodular hyperplasia, *HCC* hepatocellular carcinoma

### Qualitative assessment

**Fig. 3:**
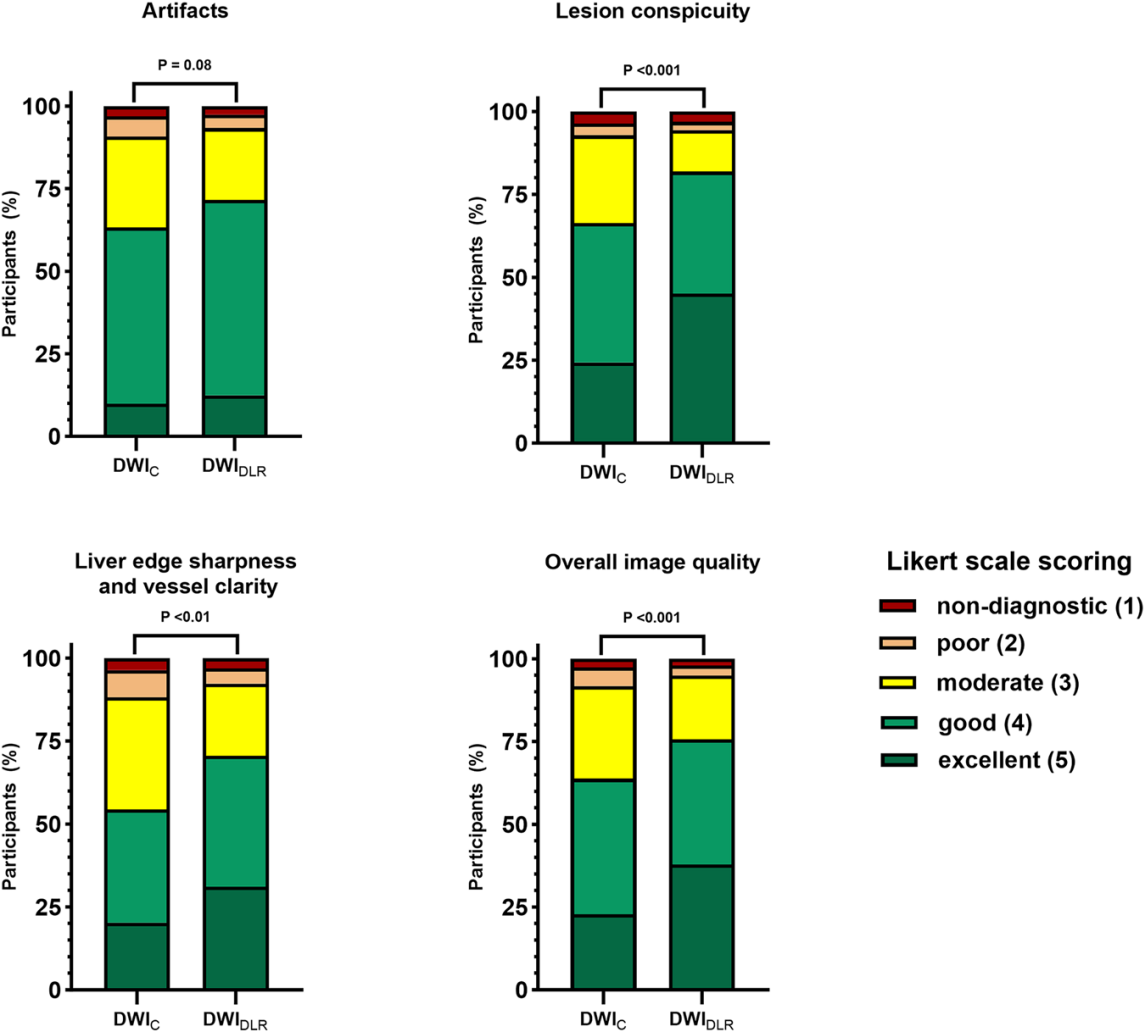
The stacked bar charts illustrate the distribution of Likert scale scores for qualitative image assessment between DWI with and without DLR. No difference was observed for artifact ratings, while qualitative scores for lesion conspicuity, liver edge sharpness, vessel clarity, and overall image quality were significantly higher with DWI using DLR

**Fig. 4:**
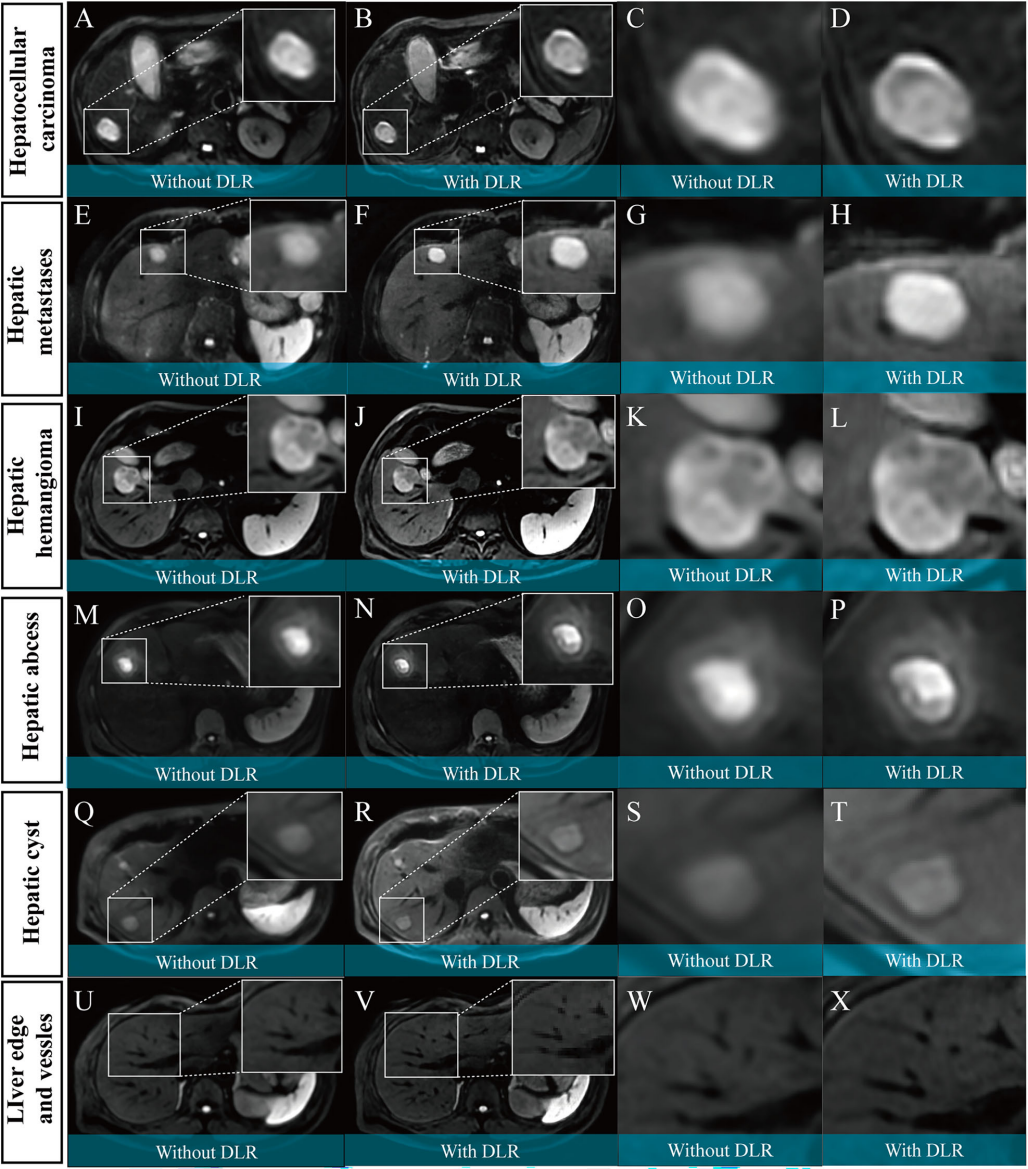
DWI images of Liver Lesions with and without DLR. The first and second columns show lesions without DLR (**A**,** E**,** I**,** M**,** Q**,** U**) and with DLR (**B**,** F**,** J**,** N**,** R**,** V**). The third and fourth columns are magnified views of the corresponding images. Lesions include hepatocellular carcinoma (**A**–**D**), hepatic metastases (**E**–**H**), hepatic hemangioma (**I**–**L**), hepatic abscess (**M**–**P**), and hepatic cyst (**Q**–**T**). The last row (**U**–**X**) shows the liver edge and vessels. The use of DLR demonstrates improved lesion visualization, enhanced edge sharpness, and better vessel clarity compared to conventional DWI

For the qualitative assessment, no significant difference was observed in the average artifact scores between DWI_DLR_ (3.61 ± 0.82) and DWI_C_ (3.84 ± 0.99, *p* = 0.08). However, DWI_DLR_ significantly outperformed DWI_C_ in lesion conspicuity (4.13 ± 0.97 vs. 3.73 ± 0.95, *p* < 0.001), liver edge sharpness and vessel clarity (3.84 ± 0.99 vs. 3.51 ± 0.98, *p* < 0.01), and overall image quality (4.01 ± 0.94 vs. 3.68 ± 0.93, *p* < 0.01). These findings suggest that DLR technology substantially improves the conspicuity of lesions, the clarity of liver edges and vessels, and overall image quality. Detailed results are presented in Table 2 and illustrated in Fig. 3. Representative images are displayed in Fig. 4.

**Table 2:** Qualitative and quantitative analysis of DWI image quality with and without DLR

Qualitative analysis (*n* = 193)	DWI without DLR	DWI with DLR	*p*-value
Artifacts	3.84 ± 0.99	3.61 ± 0.82	0.08
Lesion conspicuity	3.73 ± 0.95	4.13 ± 0.97	< 0.001
Liver edge sharpness and vessel clarity	3.51 ± 0.98	3.84 ± 0.99	< 0.001
Overall image quality	3.68 ± 0.93	4.01 ± 0.94	< 0.001
**Quantitative analysis** ***(n = 182)***			
SNR_Liver_	36.3 ± 16.1	39.6 ± 14.3	0.039
SNR_Lesion_	56.9 ± 28.6	63.5 ± 26.3	0.006
CNR	33.8 ± 27.6	39.7 ± 24.5	0.006
ERD (mm)	3.34 ± 0.39	2.38 ± 0.36	< 0.001

Data are shown as mean values  ±  deviation

*DLR* deep learning reconstruction, *SNR*_Liver_ signal-to-noise ratio of liver, *SNR*_Lesion_ signal-to-noise ratio of lesion, *CNR* contrast-to-noise ratio, *ERD* edge rise distance

### Quantitative assessment

**Fig. 5:**
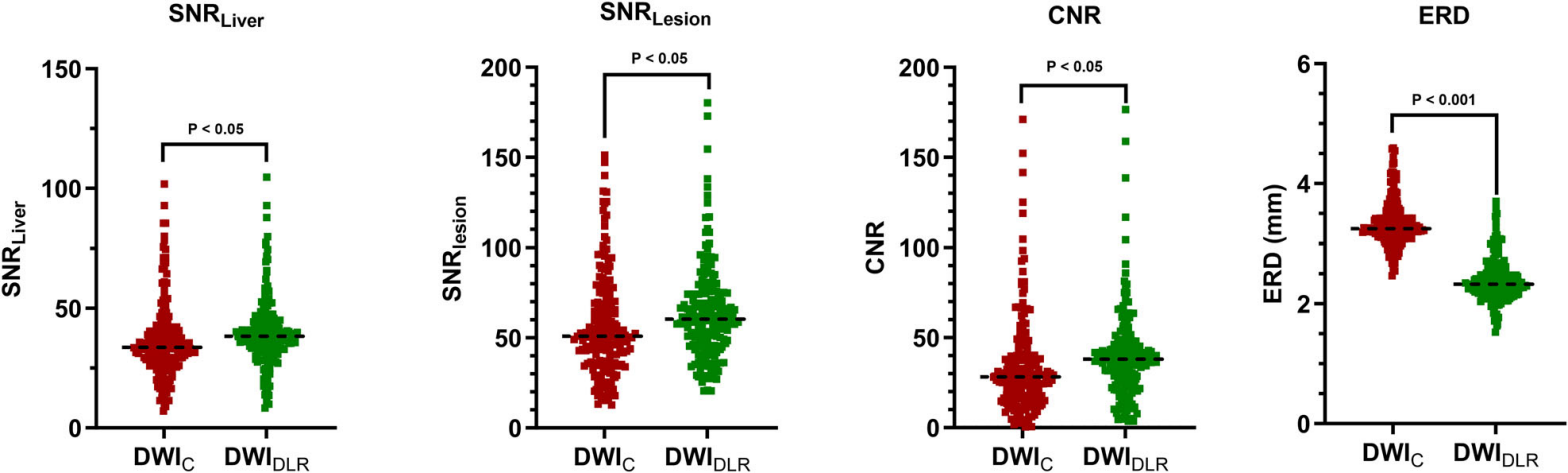
Scatter plot showing quantitative assessment metrics for each patient in DWI sequences with or without DLR. The SNR_Liver_, SNR_Lesion_, and CNR in the DWI with DLR group were higher than those in the DWI without DLR group. The edge rise distance (ERD) for DWI with DLR was lower compared to DWI without DLR. The black dashed line represents the mean

To ensure accurate quantitative assessments, 11 lesions were excluded due to poor image quality, leaving 182 lesions for analysis. The SNR_Liver_ with DLR was 39.6 ± 14.3, statistically higher than the 36.3 ± 16.1 observed in DWI_C_ (*p* < 0.05). Similarly, the SNR_Lesion_ increased to 63.5 ± 26.3 with DLR, compared to 56.9 ± 28.6 without DLR (*p* < 0.05). The CNR also improved, reaching 39.7 ± 24.5 for DWI_DLR_, compared to 33.8 ± 27.6 with DWI_C_ (*p* < 0.05). Furthermore, ERD was reduced in DLR-enhanced images, measuring 2.38 ± 0.36 mm, compared to 3.34 ± 0.39 mm in non-DLR images (*p* < 0.001). Data are shown in Table 2 and Fig. 5. The ICC for inter-reader reproducibility of all quantitative metrics was good or excellent (0.813–0.941). Data are shown in Table S3.

### Comparison of ADC values and diagnostic performance

**Fig. 6:**
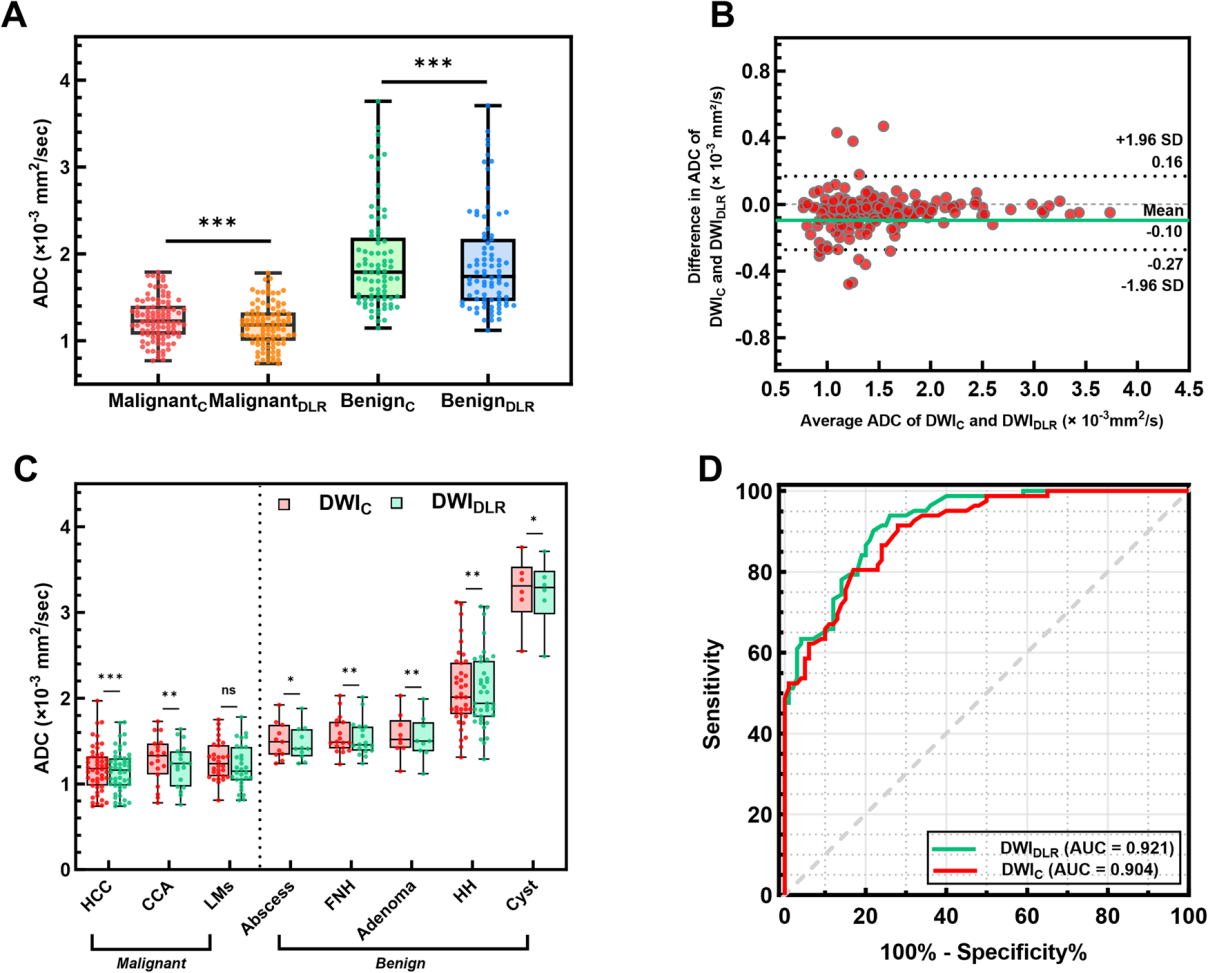
Comparison of ADC values and diagnostic performance between DWI with and without DLR. **A** Boxplots of ADC values for malignant and benign lesions with and without DLR. **B** Bland-Altman plot showing agreement of ADC values for DWI with and without DLR. **C** Boxplots comparing ADC values across different types of FLLs with and without DLR. ns, not significant, *p < 0.05, **p < 0.01, ***p < 0.001. **D** Receiver operating characteristic curves for differentiating between malignant and benign lesions using ADC values. DWIC, conventional diffusion-weighted imaging; DWIDLR, deep-learningreconstructed diffusion-weighted imaging; HCC, hepatocellular carcinoma; CCA, cholangiocarcinoma; LMs, liver metastases; FNH, focal nodular hyperplasia; HH, hepatic hemangioma

ADC values for both malignant and benign lesions were lower in DLR-enhanced sequences (Table 3). Specifically, ADC values for malignant lesions decreased to 1.18 ± 0.25 × 10^-3^ mm^2^/s with DLR from 1.25 ± 0.24 × 10^-3^ mm^2^/s without DLR (*p* < 0.001). ADC values for benign lesions decreased to 1.90 ± 0.58 with DLR from 1.92 ± 0.58 without DLR (*p* < 0.001) (Fig. 6A). The Bland-Altman plot showed a median bias of 0.095 (95% limits of agreement: -0.2734 to 0.1689 in lesions) (Fig. 6B). Further analysis using a two-way ANOVA revealed significant effects of lesion type on ADC values (*F* = 124.924, *p* < 0.001), indicating substantial differences in ADC measurements across different lesion types. However, there was no significant effect of the imaging method (DWI_DLR_ vs. DWI_C_) on ADC values (*F* = 3.273, *p* = 0.071), nor any significant interaction between imaging method and lesion type (*F *= 0.181, *p *= 0.989), suggesting that the impact of DLR on ADC values is consistent regardless of the lesion type. The ADC values for different FLLs are shown in Fig. 6C.

**Table 3:** Comparison of ADC values and diagnostic performance

		With DLR (*n* = 182)	Without DLR (*n* = 182)	*p*-value
ADC (×10^-3^ mm^2^/s)	All lesions	1.50 ± 0.56	1.55 ± 0.55	< 0.001
	Malignant lesions	1.18 ± 0.25	1.25 ± 0.24	< 0.001
	HCC (*n* = 51)	1.15 ± 0.24	1.22 ± 0.24	< 0.001
	CCA (*n* = 19)	1.21 ± 0.25	1.28 ± 0.27	0.001
	LM (*n* = 30)	1.20 ± 0.26	1.27 ± 0.22	0.051
	Benign lesions	1.90 ± 0.58	1.92 ± 0.58	< 0.001
	Abscess (*n* = 12)	1.46 ± 0.19	1.50 ± 0.21	0.032
	FNH (*n* = 18)	1.53 ± 0.21	1.57 ± 0.21	0.001
	Adenoma (*n* = 8)	1.53 ± 0.25	1.56 ± 0.25	0.001
	HH (*n* = 38)	2.08 ± 0.44	2.10 ± 0.44	0.007
	Cyst (*n* = 6)	3.22 ± 0.41	3.26 ± 0.41	0.047
	*p-*value (malignant vs. benign)	< 0.001	< 0.001	/

Data are shown as mean values  ±  deviation

*ADC* apparent diffusion coefficient, *DL* deep learning reconstruction, *HCC* hepatocellular carcinoma, *CCA* cholangiocarcinoma, *LM* liver metastases, *FNH* focal nodular hyperplasia, *HH* hepatic hemangioma, *AUC* area under the curve, *CI* confidence interval, *PPV* positive predictive values, *NPV* negative predictive values

ROC analyses showed that the AUC of DWI_DLR_ was significantly greater than that of DWI_C_ (0.921 vs. 0.904, *p* < 0.05) (Fig. 6D). As shown in Table 4, sensitivity and accuracy for detecting malignant lesions were also higher with DWI_DLR_ (90.2% and 83.5%, respectively) compared to DWI_C_ (80.5% and 81.9%, respectively). These findings indicate that DLR significantly enhances diagnostic performance in differentiating benign from malignant FLLs. To address potential confounding from high-ADC benign lesions, a subgroup analysis was conducted to differentiate malignant lesions (*n* = 100) from the most common solid benign lesions (FNH and HH combined, *n* = 56). DWI_DLR_ demonstrated improved diagnostic performance compared to DWI_C_, with a slightly higher AUC (0.946 vs. 0.928, *p* = 0.046), but no significant differences were observed in specificity, PPV, NPV, or accuracy (all *p* > 0.05). Sensitivity was identical at 96.4% (54/56) for both methods, with no statistical comparison performed. Detailed metrics are presented in Table 4, and subgroup ROC curves are provided in Supplementary Fig. S4.

**Table 4:** Diagnostic performance based on ADC

Malignant (*n* = 100) vs. Benign (*n* = 82)			
Parameters	With DLR (*n* = 182)	Without DLR (*n* = 182)	*p*-value
AUC	0.921	0.904	< 0.05
95% CI	0.885–0.957	0.863–0.945	/
Threshold ADC value (×10^-3^ mm^2^/s)	1.355	1.475	/
Sensitivity (%)	90.2 (74/82)	80.5 (66/82)	< 0.001
Specificity (%)	78 (78/100)	83 (83/100)	< 0.001
PPV (%)	77.1 (74/96)	79.5 (66/83)	0.002
NPV (%)	90.7 (78/86)	83.8 (83/99)	0.001
Accuracy (%)	83.5 (152/182)	81.9 (149/182)	< 0.001
Youden index	0.68	0.63	/
**Malignant** ***(n = 100)*** **vs. FNH** ***(n = 18)*** + **HH** ***(n = 38)***			
Parameters	With DLR (*n* = 156)	Without DLR (*n* = 156)	*p*-value
AUC	0.946	0.928	0.046
95% CI	0.914-0.977	0.891-0.966	/
Threshold ADC value (×10^-3^ mm^2^/s)	1.375	1.365	/
Sensitivity (%)	96.4 (54/56)	96.4 (54/56)	/
Specificity (%)	79 (79/100)	72 (72/100)	0.121
PPV (%)	72.0 (54/75)	65.9 (54/82)	0.927
NPV (%)	97.5 (79/81)	97.3 (72/74)	0.249
Accuracy (%)	85.3(133/156)	80.8(126/156)	0.121
Youden index	0.75	0.68	/

Data are presented as AUC values, 95% confidence intervals, threshold values, and percentages (numerator/denominator)

*ADC* apparent diffusion coefficient, *DLR* deep learning reconstruction, *FNH* focal nodular hyperplasia, *HH* hepatic hemangioma, *AUC* area under the curve, *CI* confidence interval, *PPV* positive predictive values, *NPV* negative predictive values

## Discussion

In this study, using a novel DLR algorithm, we achieved super-resolution reconstruction for liver DWI imaging, demonstrating that DWI with DLR significantly enhances liver image quality, even when acquisition time is substantially reduced. Furthermore, DWI enhanced with DLR has demonstrated robust capability in distinguishing between benign and malignant FLLs based on ADC values.

Previous DLR studies have consistently improved image quality [18–20, 29, 30], but often lacked dedicated super-resolution components to enhance fine details. For studies with super-resolution techniques like pixel-shuffle [20], the absence of multi-scale characteristics may limit their effectiveness. Our study helps address these gaps by using Adaptive CS-Net and SuperRes-Net for targeted acceleration and resolution enhancement. Adaptive CS-Net integrates compressed sensing and deep learning for multi-scale denoising and data consistency, reducing noise while preserving details. SuperRes-Net further improves quality by enhancing spatial resolution. Together, this dual CNN architecture enhances SNR and CNR compared to DWI_C_. Qualitative analysis shows that DWI_DLR_ provides superior lesion clarity, liver edge definition, and vessel delineation compared to DWI_C_, with a significant reduction in edge rise distance (ERD), leading to sharper lesion boundaries. Although both sequences showed comparable motion artifact control (*p* = 0.08, statistically non-significant), DWI_DLR_ achieved this with only half the NSA of DWI_C_ (1 vs. 2). This may be attributed to the DL model’s inherent ability to reduce ringing artifacts.

Regarding scanning efficiency, previous studies showed varied results for DWI sequences with DLR; some studies reported reduced acquisition times with DLR [20], while others found no significant change [18]. This variation was related to parameters of DWI, such as parallel imaging factors and the number of signal averages (NSA). Theoretically, reducing these two factors can shorten scan time, but may compromise image quality and SNR. We reduced NSA in DWI_DLR_ to decrease acquisition time while simultaneously improving image quality and SNR, which is primarily due to the advanced DLR algorithm used. Specifically, the sequence time for RT-DWI sequences was halved to just 1 min and 48 s. Although the actual scan time for RT-DWI may vary depending on patient respiratory cooperation, DWI_DLR_ still consistently resulted in shorter scan times.

The ADC value has been reported to reflect histological information of liver lesions and assist in differentiating benign from malignant lesions [31, 32]. Current research on the impact of DLR on liver ADC values is limited, and the existing findings are contradictory. While some studies have observed that ADC values derived from DL algorithms tend to be lower [20, 30], others have reported opposite results [29]. ADC has been reported to be affected by many factors, including the strength of the magnetic field, the protocols of the sequences, the *b*-values, the number of averages, and DLR algorithms [19, 20]. Our study found that ADC values were lower in DWI_DLR_ for both benign and malignant FLLs (both *p* < 0.001). However, the Bland-Altman analysis indicated a small median bias between the two techniques, with overall agreement within clinically acceptable limits. This suggests that, despite a slight bias, DLR-derived ADC measurements remain clinically reliable. Furthermore, two-way ANOVA revealed significant variability in ADC values across different FLL types (*F* = 124.924, *p* < 0.001) but no significant impact of imaging technique (DWI_C_ or DWI_DLR_) on ADC values (*F* = 3.273, *p* = 0.071), and no interaction between imaging method and lesion type (*F *= 0.181, *p* = 0.989). This consistency suggests that DLR maintains reliable ADC measurements across varying FLLs, supporting its potential clinical application. The underlying mechanism of DLR’s effect on ADC values remains unclear; it may be related to DLR’s influence on image noise and artifact reduction, which could affect ADC calculations. Further detailed research with larger sample sizes is necessary to clarify these effects.

Since ADC values have been widely used to differentiate benign from malignant FLLs [33–35], we further compared their diagnostic performance. The AUC for DWI_DLR_ was notably greater than that for DWI_C_, suggesting that incorporating DLR enhances the ability to distinguish malignant from benign FLLs. Additionally, the sensitivity and accuracy of DWI_DLR_ were significantly higher than DWI_C_ (both *p* < 0.001), underscoring the potential of DLR to improve overall diagnostic accuracy. However, DWI_DLR_ showed slightly lower specificity and PPV, likely due to increased false positives from the algorithm’s consistent ADC reduction, which lowers the optimal threshold and favors sensitivity over specificity at the chosen ROC point (Table 4). This does not suggest an inherent DL limitation but rather threshold-dependent effects; adjustments could optimize for higher specificity if clinically warranted. However, this finding should be interpreted cautiously, as benign lesions with higher ADC values (such as HHs and liver cysts) may have exaggerated the differences between benign and malignant groups, potentially inflating the overall diagnostic performance. A subgroup analysis excluding high-ADC lesions (e.g., cysts and abscesses) and focusing on malignant vs. combined FNH and HH partially addresses this by confirming DWI_DLR_’s enhanced performance, with a higher AUC and improved metrics (Table 4), though other performance metrics showed no significant differences, likely due to the smaller subgroup size.

While our findings highlight ADC’s utility in routine clinical practice for common FLLs, ADC contributes as a quantitative adjunct within a multiparametric workflow rather than as a stand‑alone criterion. In our everyday case mix, DLR preserved ADC-based discrimination and achieved comparable or better separation, supporting its use as part of standard reading. Potential use cases include triage of indeterminate small lesions and longitudinal surveillance, provided that acquisition and reconstruction remain stable. ADC findings should be interpreted together with morphology, dynamic enhancement, and clinical information.

Our study has several limitations. First, the sample may not fully represent the range of benign and malignant lesions due to the absence of rare or unusual diagnoses; future studies should ensure a balanced lesion type representation. Second, all imaging was performed using specific hardware and software configurations, which may limit the applicability of our results. Lastly, our study did not evaluate the performance of DLR at higher *b*-values (e.g., *b* > 1000 s/mm²); further studies are in progress to address this issue.

## Conclusions

In summary, our study demonstrated that DLR-enhanced liver DWI allows for a significant reduction in scan time while simultaneously improving image quality and enhancing diagnostic performance in differentiating benign from malignant FLLs.

## Supplementary information

ELECTRONIC SUPPLEMENTARY MATERIAL

## Data Availability

The datasets generated during the current study are not publicly available due to our institutional regulations, but are available from the corresponding author on reasonable request.

## Abbreviations

ADC: Apparent diffusion coefficient
AUCs: Areas under the ROC curves
CCA: Cholangiocarcinoma
CI: Confidence interval
CNNs: Convolutional neural networks
CNR: Contrast-to-noise ratio
CS: Compressed sensing
DLR: Deep learning reconstruction
DWI: Diffusion-weighted imaging
DWI_C_: Conventional diffusion-weighted imaging
DWI_DLR_: Deep learning reconstructed diffusion-weighted imaging
ERD: Edge rise distance
FLLs: Focal liver lesions
FNH: Focal nodular hyperplasia
HCC: Hepatocellular carcinoma
HH: Hepatic hemangioma
ICC: Intraclass correlation coefficient
LMs: Liver metastases
NSA: Number of signal averages
PI: Parallel imaging
ROC: Receiver operating characteristic
ROI: Region of interest
SNR: Signal-to-noise ratio
SuperRes-Net: Super resolution network
