# Supplementary material for: Deep learning-enhanced super-resolution diffusion-weighted liver MRI: improved image quality, diagnostic performance, and acceleration
Source: Insights Imaging. 2025 Dec 8;16:273. doi: 10.1186/s13244-025-02150-y (PMC12686321; doi:10.1186/s13244-025-02150-y)

Deep learning-enhanced super-resolution diffusion-weighted liver MRI: improved image quality, diagnostic performance, and acceleration

ELECTRONIC SUPPLEMENTARY MATERIAL

Supplementary methods

The Deep Learning Framework

The deep learning framework contains two different convolutional neural network (CNN) based reconstruction schemes: acceleration and Super-Resolution. The first CNN, Adaptive CS-Net, reconstructs images acquired with variable density undersampling patterns based on Compressed SENSE [1,2]. It is applied before coil combination to reduce noise, enhancing image quality even with accelerated acquisitions. The second CNN, Precise Image Net was designed to remove ringing artifacts and replace the traditional zero-filling approach to increase matrix size, thereby improving image sharpness. These networks, referred to as Super-Resolution networks [3,4], are trained using pairs of low- and high-resolution data with k-space crops to induce ringing. Data consistency checks ensure that the reconstructed k-space aligns with the measured data. This entire pipeline delivers images with better SNR, enhanced sharpness, increased matrix size, and reduced ringing artifacts, suitable for all 2D Cartesian acquisitions.

References

[1] N. Pezzotti, E. de Weerdt, S. Yousefi, M.S. Elmahdy, J. van Gemert, C. Schülke, et al. Adaptive-CS-Net: Fast MRI with Adaptive Intelligence, arXiv (2019), https://doi.org/10.48550/arXiv.1912.12259.

[2] N. Pezzotti, S. Yousefi, M.S. Elmahdy, J. van Gemert, C. Schülke, M. Doneva, et al. An Adaptive Intelligence Algorithm for Undersampled Knee MRI Reconstruction, arXiv (2020), https://doi.org/10.48550/arXiv.2004.07339.

[3] Y. Li, B. Sixou, F. Peyrin, A Review of the Deep Learning Methods for Medical Images Super Resolution Problems. *IRBM* 42 (2021) 120-133,

[4] C. Dong, C.C. Loy, K. He, X. Tang. Image Super-Resolution Using Deep Convolutional Networks. arXiv (2015), https://doi.org/10.48550/arXiv.1501.00092.

Supplementary tables

Supplementary Table 1. The MRI acquisition Protocol

Sequence Parameters	**T1WI**	**T2WI**	**DWI$_C$**	**DWI$_{DLR}$**
Protocol	Fast field echo with mDixon	Turbo spin echo	Echo planar imaging	Echo planar imaging
Acquisition plane	Axial	Axial	Axial	Axial
Acquisition mode	Breath-hold	Breath-hold	Respiratory-triggered	Respiratory-triggered
Field of view (mm × mm)	400 × 320	380 × 358	370 × 303	370 × 303
Matrix size	288 × 184	272 × 223	132 × 108	132 × 108
Repetition time (msec)	3.7ms	1000ms	Shortest	Shortest
Echo time (msec)	1.32ms/2.4ms	70ms	Shortest	Shortest
Parallel imaging factor*	4	2	2.4	2.4
Section thickness (mm)	2.5	6	6	6
Intersection gap (mm)	N/A	1	0.5	0.5
Acquisition time (min:sec)	0:16	0:32	3:18	1:48
b value (sec/mm^2)	N/A	N/A	0, 800	0, 800
Fat suppression	Dixon	N/A	SPAIR	SPAIR
NSA	1	1	2	1

DWI$_C$, conventional diffusion weighted imaging; DWI$_{DLR}$, deep learning reconstructed diffusion weighted imaging; SPAIR, spectral presaturation with inversion recovery; NSA, number of signal averages.

Supplementary Table 2. DWI Image quality evaluation based on the 5-point Likert scale

Factors	Score	Definition
Artifacts	1	Non-diagnostic
	2	Severe, interfering the interpretation of main anatomical component of liver
	3	Moderate, interfering the interpretation of fine anatomical component of liver
	4	Minor artifacts, not interfere with the interpretation of fine anatomical component of liver
	5	No artifact
Lesion conspicuity	1	Lesion unidentifiable
	2	No differentiation between lesion and normal anatomy
	3	Subtle lesion with poorly defined edges
	4	Well-seen lesion with poorly defined edges
	5	Well-seen lesion with well-defined edges
Liver edge sharpness and vessel clarity	1	Non-diagnostic. The liver contour and trunk of vessels are unidentifiable.
	2	Severe blurring. The liver contour and primary trunks of liver vessels are not clear.
	3	Moderate blurring. The liver contour and primary vessels is visible, but the secondary branches of liver vessels are not clear.
	4	Minimal blurring. The contour of the liver and blood vessels can be clearly identified.
	5	Sharp, no blurring.
Overall Image quality	1	Non-diagnostic
	2	Poor. Substantial deficits in image quality, which definitely impact the interpretation
	3	Moderate, with some defects potentially affecting the interpretation
	4	Good, with only minor defects that do not significantly impact image interpretation
	5	Excellent, with no noticeable defects

Supplementary Table 3. Interobserver Agreements for Quantitative Index Evaluations

Quantitative index	Method	Reader1	Reader2	ICC (95% CI)
**SNR_{Liver}**	DWI without DLR	33.6 ± 16.1	39.3 ± 16.3	0.919 (0.099-0.978)
	DWI with DLR	37.1 ± 14.4	42.0 ± 14.3	0.938 (0.006-0.968)
**SNR_{Lesion}**	DWI without DLR	57.6 ± 33.7	58.2 ± 34.2	0.941 (0.922-0.956)
	DWI with DLR	63.6 ± 27.2	63.4 ± 26.6	0.916 (0.889-0.937)
**CNR**	DWI without DLR	34.9 ± 27.8	32.7 ± 28.6	0.912 (0.884-0.934)
	DWI with DLR	40.8 ± 24.9	38.7 ± 25.4	0.894 (0.861-0.920)
**ERD**	DWI without DLR	3.34 ± 0.41	3.32 ± 0.42	0.845 (0.798-0.882)
	DWI with DLR	2.39 ± 0.38	2.38 ± 0.38	0.813 (0.757-0.857)
**ADC**	DWI with DLR	1.58 ± 0.56	1.54 ± 0.56	0.868 (0.827-0.900)
	DWI with DLR	1.48 ± 0.57	1.42 ± 0.58	0.874 (0.835-0.905)

Data are presented as mean ± standard deviation. ICC, intraclass correlation coefficient; SNR_{Liver}, signal-to-noise ratio of liver; SNR_{Lesion}, signal-to-noise ratio of lesion; CNR, contrast-to-noise ratio; ERD, edge rise distance; ADC, apparent diffusion coefficient.

Supplementary figures and figure captions

Figure S1. Graphical representation of the deep learning-based MRI reconstruction framework. FFT, Fourier fast transform; CS-NET, channel and spatial attention network.

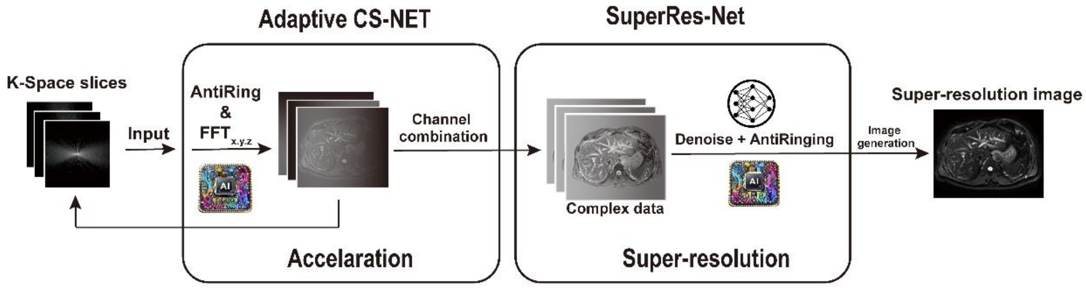

Figure S2. Diagrams show region of interest (ROI) placement. (**A**) The ROIs were placed over the lesions (arrowhead), left/right lobes of liver and erector spinae muscle. (**B**) The ROIs of lesions were copied to apparent diffusion coeficient (ADC) maps to mearsure the ADC values of corresponding area. ADC, apparent diffusion coefficient.

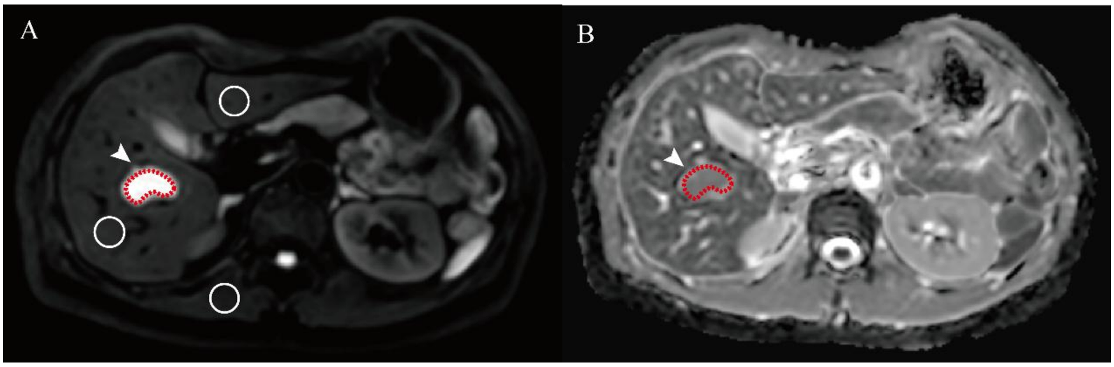

Figure S3. Comparison of acquisition time between DWI_{DLR} and DWI_C. DWI_C, conventional diffusion weighted imaging; DWI_{DLR}, deep learning reconstructed diffusion weighted imaging.

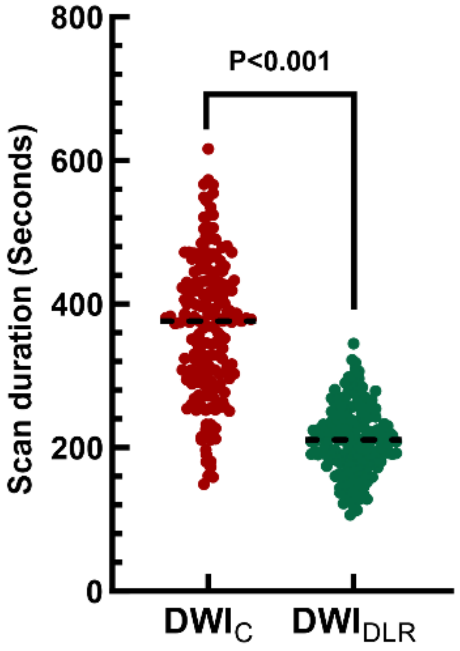

Figure S4. ROC curves for subgroup analysis comparing malignant lesions (n=100) and combined FNH+HH lesions (n=56) using DWI_{DLR} and DWI_{C}. DWI_{C}, conventional diffusion weighted imaging; DWI_{DLR}, deep learning reconstructed diffusion weighted imaging.

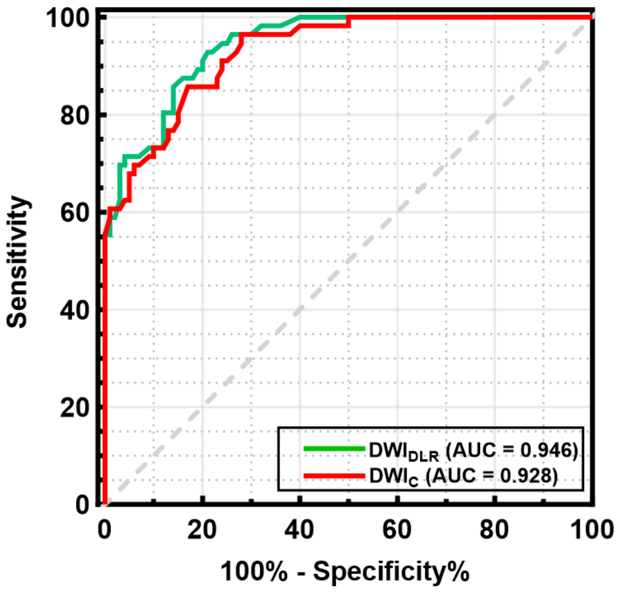

Supplement: Supplementary file 1 — ELECTRONIC SUPPLEMENTARY MATERIAL [file 13244_2025_2150_MOESM1_ESM.pdf]
